# Is digital documentation always best?

**DOI:** 10.1002/ijgo.71079

**Published:** 2026-05-16

**Authors:** Tanvi Rai, Helen Millward, Kate Siddall, Nathan S. Riddell, Amy Elsmore, Amrit Dhadda, Seren Willson, Rachel Collis, William Parry‐Smith, Peter Collins, Lisa Hinton, Sarah Bell

**Affiliations:** ^1^ Nuffield Department of Primary Care Health Sciences University of Oxford Oxford UK; ^2^ The Shrewsbury and Telford Hospitals NHS Trust Telford UK; ^3^ Cardiff and Vale University Health Board Cardiff UK; ^4^ School of Medicine, Keele University Keele UK; ^5^ NIHR Midlands Patient Safety Research Collaboration Birmingham UK

**Keywords:** clinical decision‐making, clinical documentation, electronic patient records, human factors, multidisciplinary team, obstetric emergencies, patient safety, postpartum hemorrhage

The national push toward digitization in the National Health Service (NHS)^1^ is hard to argue with. Electronic patient records (EPRs) promise efficiency, standardization, information availability, cross‐service integration, and reduced environmental impact. However, our ongoing OBS UK (Obstetric Bleeding Study UK) (www.obsuk.org) research has revealed a significant blind‐spot—the real‐time documentation of emergencies, with postpartum hemorrhage (PPH) as our exemplar. The Royal College of Midwives recommends the use of EPRs to contemporaneously record care,^2^ and NHS England aimed for up to 95% EPR implementation by March 2026.^3^ Yet, for the multidisciplinary teams (MDTs) providing emergency obstetric care across the United Kingdom, real‐time documentation using EPR systems is challenging, and as a result it is often not performed, which has important implications for patient safety.

Documentation during a PPH is difficult because the situation can evolve rapidly and require escalation to senior staff or transfer to theater. Real‐time documentation must be easy to perform and should facilitate rather than hinder care. In the past, when all members of the MDT used the same set of paper notes, a nominated scribe would log events in real time, often using a proforma to guide care and documentation. Through observations, interviews, and ongoing communication with local MDTs participating in our trial, we have identified a recurring theme: in ongoing major bleeding, staff using EPRs are forced into workarounds, jotting notes on scraps of paper towels, gloves, or scrubs, or adding entries into free‐text fields of the EPR, as the ‘correct’ format for entering these data is too fragmented, clunky, and unintuitive. In some EPRs, PPH episodes cannot even be logged until a ‘baby record’ is created, inadvertently forcing staff to suspend documentation even when PPH management is occurring concurrently with the routine newborn assessments.

EPR systems are frequently configured such that different professional groups document care on separate screens, increasing the risk that key data are missed, double‐counted, inconsistently recorded, or interpreted differently. System timeouts or internet lapses are common, necessitating staff finding time (often beyond the length of their shift) to re‐enter or finish data entry retrospectively. Smaller labor rooms leave little space for a computer terminal to be installed or even wheeled in, and in maternity units with a ‘100% paperless’ policy, this means having no structured way to record the PPH emergency as it develops. In rapid, severe PPHs, these obstacles are not minor inconveniences—they risk delaying treatment, obscuring communication, and missing critical steps. In extreme cases, it could threaten the safety of mothers and babies. If documentation is poor, and lacks the algorithmic ‘checklist’ functionality long understood to improve outcomes,^4^ it contributes not just to lapses and failures in care but also makes it impossible for reviewers investigating those cases to sufficiently reconstruct what actually happened.

Most of the 36 maternity units participating in OBS UK are either completely or mostly digital (and paperless). There are currently 20 to 25 distinct EPR systems in use nationally,^5^ with some maternity units using multiple systems, each serving separate purposes and unable to communicate with each other. The implications extend beyond PPH, and even obstetrics. All medical emergencies are time‐critical, requiring rapid, coordinated responses where situational awareness and closed‐loop communication (ensuring the intended message and action are correctly understood and completed) are vital. The literature on aviation and human factors^6^ is clear: in high‐stakes, fast‐evolving situations, visible checklists and shared documentation devices improve performance.^7^ Yet, in healthcare, despite early warnings about EPR use from other emergency settings,^8^ electronic systems are being adopted wholesale without appropriately evaluating their impact in emergencies.

The 2025 Health Service Safety Investigation report found that EPR systems can contribute to patient safety risks when they are poorly implemented, difficult to use, misaligned with the needs of staff and clinical workflows, and lack interoperability with other systems within the organization.^9^ These risks are magnified in obstetric emergencies as cases are unpredictable, compressed in time, and need urgent clinical decisions based on rapidly evolving conditions. Delayed care, missed steps, or fractured communication carry enormous consequences for women and their babies, as well as for staff and the wider system. Maternity litigation accounts for the largest share of NHS indemnity costs,^10^ and robust documentation is both a safety and a legal necessity. We are not suggesting a retreat from digitization, but consideration of the impact of the EPR systems currently in use in emergency care. Research is urgently required to understand the implications of electronic documentation in emergencies and how to optimize our systems to avoid compromising care. This mandate to shift rapidly to a paperless system in emergencies must be halted until the safety of new systems is assured.

## AUTHOR CONTRIBUTIONS

All authors were involved in the conceptualisation, analysis and, interpretation for this work. TR drafted the original version, which was critically reviewed, edited, and finally approved by all the other authors for submission.

## FUNDING INFORMATION

The OBS UK study is funded by the National Institute for Health Research (NIHR) (NIHR 152057). Point of care testing machines and consumables are provided free of charge by Haemonetics Corporation and Werfen. CSL Behring provide Fibrinogen concentrate free of charge for the study. These funders had no role in study design, analysis, interpretation or writing of the paper. The views expressed are those of the authors and not necessarily those of the NIHR.

## CONFLICT OF INTEREST STATEMENT

SB has received research support from CSL Behring, Haemonetics and Werfen. She has acted as a paid consultant to CSL Behring and received lecture honoraria from Werfen. PC has received research support from CSL Behring, Haemonetics and Werfen. He has acted as a paid consultant to Grifols, CSL Behring and Werfen. RC has received research support from CSL Behring, Haemonetics, Werfen. NSR has received travel reimbursement from Haemonetics. All other authors have declared that no competing interests exist. OBS UK receives research support from Werfen, Haemonetics Corps and CSL Behring.

## Data Availability

Data sharing is not applicable to this article as no data sets were generated or analyzed during the current study.
